# Potential of Essential Oils from Different Mint Species Against Multidrug-Resistant *Escherichia coli* Strains Isolated from Clinical Cases in Poultry

**DOI:** 10.3390/ijms262311263

**Published:** 2025-11-21

**Authors:** Michalina Adaszyńska-Skwirzyńska, Sławomir Zych, Małgorzata Dzięcioł, Paweł Konieczka, Barbara Kowalik, Dorota Witkowska, Mateusz Bucław

**Affiliations:** 1 Department of Monogastric Animal Sciences, Faculty of Biotechnology and Animal Husbandry, West Pomeranian University of Technology in Szczecin, Janickiego St. 29, 71-270 Szczecin, Poland; mateusz.buclaw@zut.edu.pl; 2 Department of Microbiology and Biotechnology, Faculty of Biotechnology and Animal Husbandry, West Pomeranian University of Technology in Szczecin, Piastów Ave. 45, 70-310 Szczecin, Poland; slawomir.zych@zut.edu.pl; 3 Department of Organic Chemical Technology and Polymer Materials, Faculty of Chemical Technology and Engineering, West Pomeranian University of Technology in Szczecin, Piastów Ave. 42, 71-065 Szczecin, Poland; malgorzata.dzieciol@zut.edu.pl; 4 Department of Poultry Science and Apiculture, University of Warmia and Mazury in Olsztyn, Oczapowskiego St. 5, 10-719 Olsztyn, Poland; pawel.konieczka@uwm.edu.pl; 5 The Kielanowski Institute of Animal Physiology and Nutrition, Polish Academy of Sciences, Instytucka St. 3, 05-110 Jabłonna, Poland; b.kowalik@ifzz.pl; 6 Department of Animal Welfare and Research, Faculty of Animal Bioengineering, University of Warmia and Mazury in Olsztyn, Oczapowskiego St. 5, 10-719 Olsztyn, Poland; dorota.witkowska@uwm.edu.pl

**Keywords:** antibacterial activity, antibiotic resistance, avian colibacillosis, *Escherichia coli*, mint essential oils

## Abstract

The study presents the potential application of three essential oils (EOs) obtained from various mint species: peppermint (*Mentha piperita* L.), spearmint (*Mentha spicata* L.), and pennyroyal (*Mentha pulegium* L.), as alternative agents against multidrug-resistant *Escherichia coli* strains isolated from poultry. The aim of research was to determine the effectiveness of EOs from these mint species and their main chemical components (menthol, menthone, carvone, pulegone) against 19 clinical *E. coli* strains with varying drug susceptibility and the reference strain *E. coli* ATCC 25922. GC-MS analysis revealed a unique chemical profile for each mint species. In *M. piperita*, menthol (35.14%), trans-menthone (23.11%), and menthyl acetate (6.96%) were the dominant compounds. In *M. spicata*, the main components were carvone (58.61%) and 1,8-cineole (18.74%), while in *M. pulegium*, pulegone was the dominant ingredient (76.54%). Antibacterial activity tests showed that all tested *E. coli* strains, both susceptible and multidrug-resistant, were sensitive to the EOs. The strongest antibacterial activity was observed for peppermint oil (Minimal Inhibitory Concentration–MIC 0.5–2.0% *v*/*v*), which was attributed to its high menthol content. Menthol was also the most effective single component (MIC 0.125–0.5% *v*/*v*). The essential oils containing carvone and pulegone showed lower activity (MIC ≥ 2% *v*/*v*). Research indicates that mint EOs, particularly those rich in menthol, represent a promising alternative or complement in the treatment of infections caused by multidrug-resistant *E. coli* strains.

## 1. Introduction

Species of the genus *Mentha* belong to the most popular essential oil (EO) plants in the family of *Lamiaceae* [1]. Their medicinal properties are primarily attributed to their EO content, which explains their wide applications in the veterinary, pharmaceutical, cosmetic, and food industries [2,3]. In the global market, EOs from mint species rank second in production volume, just behind citrus species [4]. As of 2020, India accounts for approximately 80% of the world’s peppermint production, with an output of 37,000–40,000 tons [4,5,6]. The wide genetic diversity of *Mentha* spp., together with their propensity for intraspecific hybridization, has yielded many botanical, regional, and cultivated varieties [5,6,7,8]. Despite long-standing popularity, detailed investigations into mint’s biological effects are ongoing.

Available literature suggests that mint EOs may serve as an alternative for controlling pathogenic bacteria that have developed resistance to multiple antibiotics [9,10,11,12]. Antibiotic and chemotherapeutic resistance in bacteria is a multifaceted problem affecting both animal and human health, representing a leading challenge of the 21st century. For at least a decade, the use of preparations containing biologically active compounds with effects similar to antibiotic growth promoters, banned by the European Union, has been promoted in animal production and the feed industry. Consequently, for many years there has been considerable interest in the potential use of plant secondary metabolites, including EOs, as substances with antibacterial and immunostimulatory properties [1,2,8,9,13,14]. EOs are liquids with hydrophobic properties because they are composed primarily of nonpolar organic compounds. However, they are very good soluble in fats and some organic solvents (e.g., ethyl alcohol). Due to their poor water solubility, limitations arise in medicinal formulations, often requiring the use of emulsifiers or solubilizers [2]. Numerous studies have been conducted in this area, demonstrating that the activity of EOs enables their use in animal nutrition and veterinary prophylaxis. Consequently, the production of preparations for poultry and other monogastric animals containing EOs with strong bacteriostatic activity is increasing each year. Moreover, an undeniable advantage of these preparations is that they can be administered together with vaccines and do not require a withdrawal period, which improves the safety of food products. EOs and their main components are used as feed and water additives, as well as for maintaining hygiene in facilities, for example through fogging or inhalation [15]. Currently, as microbial resistance to antibiotics and chemotherapeutics poses serious veterinary and economic problems, natural alternatives are being sought to replace or support synthetic agents.

Antibiotic resistance involves several closely interconnected areas, including animal and human health, food, wastewater, and the environment. European Union resolutions on increasing antimicrobial resistance, including antibiotic resistance, such as Regulation (EU) 2019/6 of 11 December 2018 [16], on veterinary medicinal products are central to the “One Health” approach. This concept recognizes the close interconnection between human and animal health, and the potential for diseases to be transmitted between them, making it necessary to coordinate actions in both populations. The “One Health” approach also includes the environment, which is another factor connecting humans and animals and likely represents a potential source of new antimicrobial-resistant microorganisms.

Colibacillosis is a disease caused by avian pathogenic *Escherichia coli* (APEC) isolates which results in significant mortality in poultry [17,18]. Since 2014, Poland has been the largest producer of poultry meat in Europe, and APEC in birds causes a range of extraintestinal infections, whose treatment is increasingly difficult due to the emergence of multidrug-resistant strains. *E. coli* strains are currently classified as so-called alert pathogens, producing extended-spectrum beta-lactamases (ESBL) [17,18,19,20]. They hydrolyze all antibiotics from the penicillin, monobactam, and cephalosporin groups, including those with a broad spectrum of activity. It has been shown that the use of clavulanic acid, sulbactam, or tazobactam as beta-lactamase inhibitors not always enhance the effectiveness of antibiotics in treating *E. coli* infections. Considering that antibiotic resistance genes can be easily transferred even between different bacterial species, these strains can pose a direct or indirect threat to humans as consumers of poultry products [21,22,23]. Meat is a likely route for transmission of antibiotic-resistant bacteria to humans, including strains resistant to multiple antibiotic classes [21,22]. Menck-Costa et al. [22] indicate that commercial chicken meat can harbor ESBL-producing *E. coli*, underscoring the need for judicious antibiotic use in food animals. Consequently, phytobiotics, including EOs, are being explored as alternative antimicrobials [19,24,25]. Despite extensive research on their broad and complex effects on microorganisms, further research is needed into the antibacterial activity of EOs against clinical strains, particularly in the context of their potential widespread use in treating infectious diseases [10,24,25].

Strategies of drug resistance fighting include research in various areas such as genetic improvement of animals to identify markers associated with increased innate resistance to pathogens, searching for new antimicrobial agents, determining the role of bacteria found in animal husbandry in the transfer of antibiotic resistance to human bacterial flora and the associated potential risk. Current alternatives being implemented involve plant extracts, bacteriophages and their lytic enzymes, new vaccines, and bactericidal/bacteriostatic proteins or peptides produced by various organisms [15,26,27]. Another strategy involves the use of probiotic strains of intestinal bacteria (*Lactobacillus* spp., *Bifidobacter* spp., *Bacillus subtilis*, and *Pediococcus acidilactici*) while simultaneously eliminating pathogenic strains. Many studies, especially conducted on farm animals, have confirmed that oral administration of probiotic strains significantly reduces the occurrence and colonization of *Salmonella Enteritidis*, *S. Enterica*, *S. Typhimurium*, *Campylobacter* spp., *Clostridium perfringens*, *Enterococcus* spp., APEC, and pathogenic *E. coli* in the intestines of poultry, pigs, and cattle [28]. Additionally, improving production practices, animal welfare, biosecurity, sanitation, and reducing antibiotic overuse are central measures against antibiotic resistance [29,30].

The aim of the study was to evaluate the efficacy of EOs from three *Mentha* species with different chemotypes, as well as their main chemical components (menthol, menthone, carvone, and pulegone), in controlling colibacillosis in poultry, using the reference *E. coli* strain ATCC 25922 (human strain, antibiotic-sensitive) and clinical strains with varying antibiotic susceptibility.

## 2. Results

Chromatographic analysis of essential oils (GC-MS) was carried out to identify compounds characteristic of the genus *Mentha* and to determine differences between its individual species. The Total Ion Chromatogram of EOs and mass spectra of their main components are collected in the “Supplementary Materials” section (Figures S1–S6). Table 1 presents the chemical composition of EOs from different *Mentha* species. A total of 36 chemical compounds were identified in the three *Mentha* species. In peppermint (*M. piperita* L.), the main chemical compounds were menthol (35.14%) and *trans*-menthone (23.11%). In spearmint (*M. spicata* L.), the main constituents were carvone (58.61%), 1,8-cineole (18.74%), and cis-dihydrocarvone (3.09%). In pennyroyal (*M. pulegium* L.), the most abundant compounds were pulegone (76.54%) and trans-menthone (6.60%).

Each of the mint species studied had a unique chemical profile, which directly influenced its antibacterial activity. *Mentha piperita* is rich in menthol and menthone, *Mentha spicata* in carvone, and *Mentha pulegium* in pulegone. These main components are responsible for the characteristic aroma, taste, and biological properties of each *Mentha* species.

The sensitivity of *E. coli* strains to EOs is presented in Table 2. The EOs tested and their main chemical components exerted variable antibacterial effects, depending on their chemical composition. Both the reference strain (ATCC 25922) and all clinical strains (1–19) were susceptible to the EOs. Peppermint oil was the most active antibacterial agent, with its high menthol content effectively inhibiting the growth of all tested strains (MIC 0.5–2.0% *v*/*v*). In turn, the EOs dominated by carvone (spearmint) or pulegone (pennyroyal), showed a markedly lower antibacterial activity against *E. coli* (MIC ≥ 2% *v*/*v*). All strains tested, including both antimicrobial-sensitive isolates and multidrug-resistant strains, had MIC values for the EOs ranging from 0.5% to 4% (*v*/*v*). Among the main chemical components of the EOs, the multidrug-resistant strains were most susceptible to menthol, with a minimum inhibitory concentration (MIC) ranging from 0.125% to 0.5% (*v*/*v*). In contrast, the MIC of this compound for antibiotic-sensitive strains ranged from 0.125% to 0.25% *v*/*v*, which indicated only slightly lower activity of this compound against some resistant strains. The results expressed in mg/mL are summarized in Table S2 in the “Supplementary Material” section.

**Figure 1:**
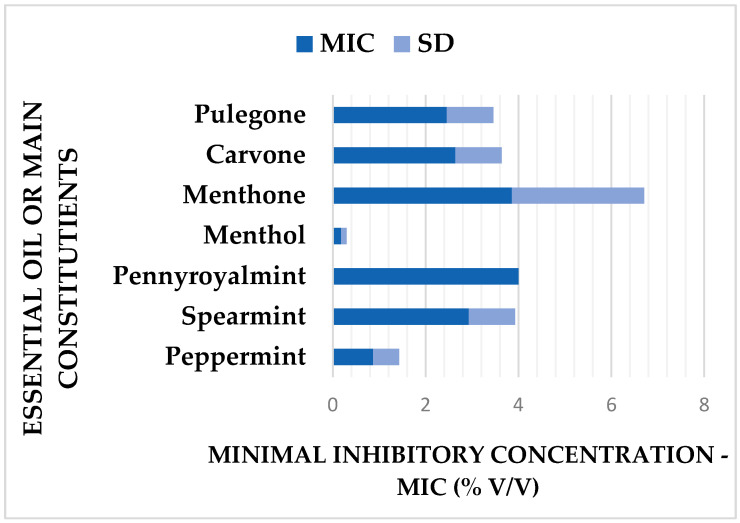
Mean minimum inhibitory concentration (MIC) values (% *v*/*v*) of EOs with standard deviation (SD, *n* = 3) from different *Mentha* species and their main constituents against 20 *Escherichia coli* strains with varying antibiotic susceptibility.

Figure 1 illustrates the mean MIC values (% *v*/*v*) of EOs from different *Mentha* species and their main components. Among the main compounds, menthol demonstrated the strongest activity with an average MIC of 0.18% (*v*/*v*). In contrast, menthone had the weakest activity, with an average MIC value of 3.86% (*v*/*v*) for all tested strains. The most potent EO was peppermint oil (MIC = 0.87% *v*/*v*), while the lowest activity was observed for pennyroyal oil (MIC = 4.0% *v*/*v*).

Highly significant differences in MIC values were observed between the three *Mentha* species. The lowest MIC values, corresponding to the highest antimicrobial activity, were recorded for peppermint. Significantly higher MIC values (*p* < 0.05) were observed for both pennyroyal and spearmint oils, with no significant difference (*p* > 0.05) in MIC between these latter two (Table 3).

**Figure 2:**
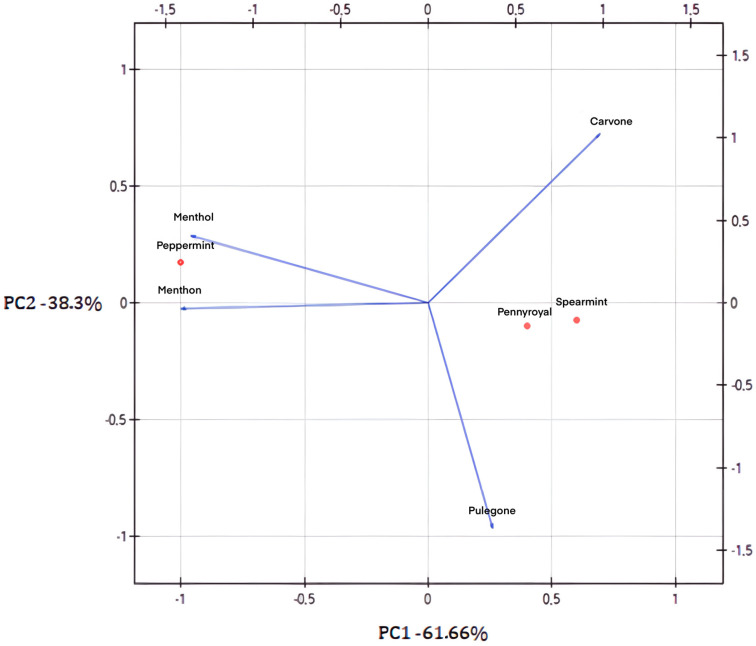
Principal component analysis (PCA) plot for essential oils from three *Mentha* species and their main components (peppermint—*Menta piperita* EO; spearmint—*Mentha spicata* EO; pennyroyal—*Mentha pulegium* EO). The vectors displayed represent the eigenvectors of the covariance matrix.

GC-MS analysis provided comprehensive information on the primary composition of *Mentha* EOs, allowing the identification and confirmation of the specific chemotype for each mint species. Analysis of the antibacterial activity results for the individual EOs and their main components, in relation to the composition of the tested oils from various mint varieties, indicates that chemotypes with the highest possible menthol content should have the greatest antibacterial potential against pathogenic *E. coli* strains. PCA (Figure 2) indicated differences between the major components of EOs from the *Mentha* species under study. Considering the projection of parameters onto the plane based on the first two components, the first component (explaining 61.66% of the variance) characterizes the menthol chemotype of mint, while the second component (38.34% of the variance) reflects the contribution of other active compounds.

## 3. Discussion

EOs contain up to several hundred chemical compounds, mainly terpenoids, divided into mono-, sesqui-, di-, tri-, tetra-, and polyterpenoids [15,31]—which underlie their diverse biological effects, often governed by the dominant compound [32,33,34]. Mint EOs are volatile mixtures ideally characterized by GC–MS, and the EOs analyzed in the study were rich in oxygenated monoterpenes, including menthol, carvone, and pulegone. GC–MS profiling confirmed significant chemical variability among *Mentha* species, in agreement with literature data [1,8,32,33,34,35,36,37]. However, there are reports in which the proportions of individual oil components differ from those used in the present study. For example, a study by Hudz et al. [38] reported a menthol content of 40.7% in *M. piperita*, while Saharkhiz et al. [39] found levels as high as 53.28%. Marwa et al. [40] reported 46.32% menthol and only 7.42% menthone. Other analyses revealed much broader ranges, with Orav et al. [41] reporting menthone from 11.2% to 45.6% and menthol from 1.5% to 39.5%. In the caraway chemotype (*M. spicata*), where carvone (58.61%) and 1,8-cineole (18.74%) were dominant in the present study, other researchers reported carvone content ranging from 40.8% [36] to as high as 78.8% [42]. In *M. pulegium* EO Aimad et al. [43] reported 76.35% of pulegone, a result nearly identical to that obtained in the current work. On the other hand, Luís et al. [44] determined pulegone at 88.64%, while El-Ghorab et al. [45] recorded only 43.5%. Such significant variations may result from the specific variety of a given mint chemotype or its cultivation conditions, which favor the biosynthesis of particular compounds [9]. Observed differences in composition may be due to such factors as place of cultivation, weather conditions, type of soil, fertilizers used, age of plant, time and method of harvesting, plant material drying conditions, and EOs producing method [46].

In poultry production, antibiotics are still used therapeutically and historically for prophylaxis. Misuse or overuse of antimicrobials exerts selective pressure favoring resistant organisms that outcompete susceptible microbes, leading to persistence and spread of resistance traits in the environment [47]. Currently, significant attention is being paid to measures aimed at reducing disease incidence by increasing bird immunity and ensuring a high level of biosecurity on farms. Searching for alternative treatments and creating conditions aimed at reducing the occurrence of infections are necessary paths to follow in order to maintain the antimicrobial effectiveness of antibiotics and reduce the problem of drug resistance [30,47].

Clinical *E. coli* strains pose a threat to animals and humans due to high resistance to antibiotics and chemotherapeutic agents. According to the EFSA report [48], *E. coli* isolated from domestic chickens in Poland show the highest resistance to antibiotics. The antibacterial activity of EOs depends not only on their type and applied concentration but also on the activity of their individual components. Understanding the relationship between the chemical composition of EOs and their antibacterial activity is important due to the potential use of EO-based preparations as natural agents against *E. coli*. Clinical strains show high resistance to antibiotics and chemotherapeutic agents [49]. Recent studies indicate that Gram-negative bacteria are more resistant to EOs due to the presence of a hydrophilic polysaccharide chain that forms a barrier against the hydrophobic molecules of EOs [24,50]. Kunicka and Kalemba [51] proposed an activity hierarchy of EO constituents: phenols > aldehydes > ketones > alcohols > esters > ethers > hydrocarbons. The mint oils used in our study contained high levels of ketones (carvone, menthone, pulegone) and monoterpene alcohols (menthol). The analyses identified menthol as the most potent antibacterial constituent of the EOs, which determined the biological activity of peppermint oil. This is consistent with literature data confirming the strong antibacterial properties of menthol [32,33,51,52,53]. Other researchers suggest that menthol may affect bacterial cell membrane properties by increasing permeability, leading to leakage of cytoplasmic contents and ultimately bacterial cell death [52]. Moreover, researchers suggest that menthol may be effective against tetracycline-resistant *E. coli* strains by altering their cell membrane properties [52,54]. This indicates its potential as an adjuvant in the treatment of infections caused by drug-resistant strains. Carvone, the main constituent of *M. spicata*, showed moderate antibacterial activity in our study. Literature data demonstrate that this compound may also act on the bacterial cell membrane, disrupting its integrity [55]. Some studies suggest that carvone shows weaker activity against *E. coli* compared to other pathogens, but its synergistic effects with other compounds and antibiotics may improve its effectiveness [54]. Pulegone, the dominant compound in *M. pulegium* (pennyroyal), showed the lowest antibacterial activity among the oil constituents. Pulegone is a monoterpene ketone that occurs in two enantiomeric forms. The (R)-(+)-enantiomer of pulegone is more common in nature and has been associated with hepatotoxicity and pulmonary toxicity [56]. Anderson et al. [57] reported in a human study that ingestion of more than 10 mL of *M. pulegium* EO caused moderate toxicity. Eighteen cases of hepatotoxicity were confirmed, along with instances of gastritis and mild neurotoxic effects.

The antibacterial properties of EOs have been empirically confirmed in numerous studies, although their exact mode of action remains unclear [32,33,50,53]. This activity may result from changes in the permeability of the bacterial cell membrane. It has been shown that certain EO components, such as carvacrol, which is found in high concentrations (>30%) in thyme oil, can permeabilize and depolarize the cytoplasmic membrane of *E. coli*, facilitating antibiotic entry into the bacterial cell [57]. It has been demonstrated that EOs can affect both the bacterial cell surface and the cytoplasm. The hydrophobic nature of EOs disrupts bacterial structures, increasing membrane permeability. This is critical because the membrane has many functions, including maintaining cellular energy status, transporting dissolved substances, and regulating metabolism. This suggests that the antimicrobial mechanisms of EOs may involve cell wall degradation, cytoplasm coagulation, leakage of cellular contents, and a reduction in the proton-motive force [22,27,30,58,59].

Dorman et al. [60] tested 14 EO compounds against 25 bacterial strains and confirmed that monoterpenoids and sesquiterpenes exhibit strong antimicrobial activity against most of the strains tested. The antibacterial potential of menthol against *E. coli* was also confirmed by Trombetta et al. [61]. Researchers have determined that the hydroxyl group present in a specific compound can contribute to its antimicrobial activity. Lopez-Romero et al. [62] evaluated the antibacterial activity and mechanisms of action of EO components, including carvacrol, carvone, citronellol, and citronellal, against *E. coli*. They found that citronellol showed the strongest activity, disrupting cell membrane integrity and surface charge, and subsequently causing leakage of K^+^ ions. Another study reported that two pentacyclic triterpenes, α-amyrin and ursolic acid, disrupted the *E. coli* cell membrane [63]. Menthol shows significant antimicrobial activity against Gram-negative bacteria (including *E. coli*) through several mechanisms. The antibacterial effects of menthol are primarily attributed to its ability to disrupt bacterial cell membranes resulting in alterations of membrane permeability and in leakage of intracellular materials [55]. Menthol is believed to have the ability to penetrate the fatty acid chains in the lipid bilayer, thereby disrupting the lipid arrangement and membrane fluidity, which in turn leads to noticeable structural and morphological changes on the cell surface [64]. Research by Azmi et al. [65] confirmed that menthol is a great hydrogen bond acceptor, and various fatty acids act as hydrogen bond donors. In addition, the study performed by Turcheniuk et al. [66] found that menthol-modified nanodiamond particles have a significant inhibitory effect on the biofilm formation of both Gram-positive bacteria (e.g., *Staphylococcus aureus*) and Gram-negative bacteria (e.g., *E. coli*). Landau and Shapira [67] report that precultivation of enterohemorrhagic *E. coli* strains in subinhibitory concentrations of menthol elevates up to 16-fold their resistance to menthol and expression of the *cpsB10* gene encoding one of the enzymes responsible for colanic acid which elevated mucoidity of colony. However, mucoidity reduced curli and biofilm formation and this suggests a general reduction in bacterial virulence following adaptation to menthol. The antibacterial mechanism of EOs containing menthone was rarely reported. Menthone exhibited antibacterial activities against methicillin resistant *S. aureus* (MRSA) where the depolarizing membrane potential and disrupting bacterial membrane integrity suggested that cell membrane, similar to menthol, might also be the target of menthone [68]. Similar observations apply to carvone. Cytoplasmic membrane permeabilization was observed by Lopez-Romero et al. [62] based on the uptake of propidium iodide (PI), a nucleic acid stain, to which intact cell membrane is usually impermeable. For carvone, the percentage of *E. coli* cells stained with PI after 1 h of treatment (at corresponding MIC) was 35%. The values of the electron donor component significantly increased (*p* < 0.05) after treatment with carvone. In addition, significant changes in the cellular surface charge of *E. coli* (*p* < 0.05) were observed after exposure to carvone. The so-called zeta potential values of *E. coli* became less negative after contact with carvone (−18.46 mV in comparison to control at the level of −22.78 mV). At physiological conditions, bacterial cells have a negative surface charge due to the presence of anionic groups. In the study of Gong et al. [69], pulegone had a biofilm inhibitory rate of 52.36% in multi-drug resistant *E. coli* K1 strain. Moreover, expression of the *pgaA*, *pgaB*, *pgaC* and *pgaD* genes (the *pgaABCD* operon for PNAG) was significantly down-regulated after treatment with 23.68 mg/mL of pulegone (*p* < 0.05). Poly-β-1,6-N-acetyl-D-glucosamine (PNAG) is an essential factor for biofilm formation in *E. coli* as it mediates cell-to-cell and cell-to-surface adhesion in biofilms. The cell membrane integrity was evaluated in the study of Farhanghi et al. [70] by measuring the release of cell constituents (nucleic acids and proteins) into the supernatant. However, pulegone at MIC 0.6% *v*/*v* did not induce significant cell constituents’ release in *S. aureus*. Such activity on Gram-negative bacteria is still unknown. The results of our studies clearly show that among tested mint EOs the most effective against all *E. coli* strains was peppermint (MIC 0.5–2.0% *v*/*v*), characterized by the highest content of menthol (about 35%). The activity of two remaining essential oils, with very low menthol content (spearmint EO, <2%) or no menthol content (pennyroyal EO), was significantly lower. This fact, as well as the outstanding activity results obtained for menthol standard against both sensitive and resistant strains (MIC 0.125–0.5% *v*/*v*) may suggest that the high content of this compound is a significant dominant factor in terms of effectiveness against *E. coli*. Our findings are consistent with those presented by other authors, who compared the antimicrobial activity of the EOs of two mint species: peppermint from Slovakia and spearmint from Italy. Their studies showed that an *E. coli* strain isolated from poultry was more sensitive to peppermint EO, containing 49.3% menthol and 22.4% menthone as the main components, than to spearmint EO, where the main component was carvone (62.4%) and the menthol content was negligible (0.2%) [71].

## 4. Materials and Methods

### 4.1. Essential Oils and Their Main Chemical Constituents

Commercial essential oils (EOs) were used to assess the activity of oils obtained from three different mint species: peppermint (*M. piperita* L.), spearmint (*M. spicata* L.), and pennyroyal (*M. pulegium* L.) (Now^®^ Foods, Bloomingdale, IL, USA). According to the manufacturer, the oils were obtained from the aerial plant parts (leafy stems and inflorescences) by steam distillation. Producers declare their high quality assured by strict cooperation with growers and suppliers to safeguard the quality of the raw materials. Moreover, each batch of particular EO is tested and confirmed for compliance with the Now^®^ Foods in-hose standards. Additionally, four major chemical compounds defining the chemotype of these mint EOs were also used in studies, as they may potentially determine their activity. The following analytical standards were used: menthol (99.9% purity) and menthone (≥81%) for peppermint EO, carvone (>93%) for spearmint EO, and pulegone (98%) for pennyroyal EO (Sigma-Aldrich, Saint Louis, MO, USA).

### 4.2. Analysis of EO Chemical Composition

Gas chromatography analyses of the EOs were performed using a 6890N gas chromatograph (Agilent Technologies, Palo Alto, CA, USA) coupled with a 5973 Network mass selective detector and equipped with a 7683 Series automatic sample injector (Agilent Technologies). For analysis, the sample was prepared by dissolving 0.02 mL of the EO in 1.0 mL of acetone. Each sample was injected and analyzed three times, and the results obtained were averaged and given with the standard deviation calculated. The chromatographic conditions were determined experimentally to achieve optimal separation of the analytes. An HP-5MSI capillary column (30 m length, 0.25 mm internal diameter, 0.25 μm film thickness) was used. The separation of the EOs constituents was achieved using the optimized temperature program (starting temperature: 50 °C, final temperature: 290 °C, ramp rate: 4 °C/min.) Helium was applied as the carrier gas at a constant flow rate of 1.2 mL/min.

Samples with a volume of 3 µL were injected in split mode (10:1). Analyses were conducted in scan mode over an m/z range of 20–500. Data acquisition and processing were performed using ChemStation (Agilent Technologies). Compounds in the samples were identified by comparing their mass spectra with reference spectra from the NIST 04 library and by means of calculated retention indices. Compounds with a mass spectral match factor above 95% compared to the reference library spectra were considered. To confirm compound identification, retention indices were calculated and compared with literature data [72]. Retention times of the respective *n*-alkanes were determined by analyzing an *n*-alkane standard (C7–C30, Supelco, Bellefonte, PA, USA) under the same chromatographic conditions. Subsequently, retention indices were calculated using the following formula:

(1) $${LRI}_{calc.}=100n+100\frac{t_{x}-t_{n}}{t_{n+1}-t_{n}}$$

where LRI_calc._ is the retention index calculated from the chromatographic data of OEs and standard of n-alkanes; t_x_, t_n_, t_n+1_ are the retention times of identified compound (x) and of n-alkanes eluted directly before (n) and after this compound (n + 1) [min].

Quantitative analysis was performed using the area normalization method. The relative contents of the particular compounds were obtained as the percentage of their peak areas in the total area of all separated peaks.

### 4.3. Bacterial Strains

The activity of three mint EOs and four major chemotype-defining compounds was tested against 19 clinical strains of Gram-negative *Escherichia coli*. The strains were isolated in 2021–2022 from clinical cases of omphalitis and yolk sac infection in one-day-old broiler chicks at a veterinary diagnostic laboratory in Szczecin, Poland. The last twentieth strain was the control reference strain *E. coli* ATCC 25922 (WDCM 00013; KWIK-STIK™ Plus, Microbiologics, St. Cloud, MN, USA). The drug resistance data were obtained from official test reports: strains numbered 1 to 10 were classified as multidrug-resistant, while strains numbered 11 to 19 and the reference (20) *E. coli* strain ATCC 25922 were susceptible to the antimicrobial agents used in poultry treatment (Table S1 in the “Supplementary Material” section). However, there is no information on the potential APEC serotypes. The susceptibility of the bacterial strains to recommended antibiotics and chemotherapeutics was determined using the disk diffusion method and verified according to CLSI guidelines [73,74]. The isolates were preserved in ViaBank™ bacterial storage systems (MWE, Medical Wire & Equipment, Potley Lane, Corsham, England) and stored < −20 °C until EO activity testing. Each strain was sequentially revived on Columbia agar supplemented with 5% sheep blood (Graso, Starogard Gdański, Poland) 24 h before inoculating the test plates, and incubated at 37 °C ± 1 °C.

### 4.4. Antibacterial Activity Assessment

Each EO, as well as all reference substances, was initially diluted in acetonitrile (LiChrosolv^®^, Supelco, Merck KGaH, Darmstadt, Germany) to a working concentration of 40% *v*/*v*. The antibacterial activity of the EOs, reference compounds, and pure controls was determined using the minimum inhibitory concentration (MIC) method on sterile 96-well plates with lids (Wuxi Nest Biotechnology, Wuxi, China). Mueller-Hinton broth (MHB) (GRASO, Gdańsk, Poland) was used as the culture medium. In the first well row, 180 µL of MHB was added, followed by 20 µL of the dilution of each EO or analytical standard, resulting in the initial 4% *v*/*v* concentration of the test substance in a horizontal arrangement on the plate. The contents were thoroughly mixed using a multichannel pipette, and 100 µL of the prepared first dilution was transferred to the next row containing 100 µL of MHB and mixed thoroughly. This procedure was repeated for subsequent rows, generating a dilution gradient on the plate from 4% to 0.06% *v*/*v*. The last row served as positive and negative controls, where all wells containing only 100 µL of MHB without any antimicrobial agent. However, to exclude the negative influence of acetonitrile on bacterial growth, the positive control wells contained an addition of 5% acetonitrile. The prepared test plate was inoculated by adding 10 µL of the selected *E. coli* strain, previously standardized to 0.5 McFarland and then diluted 10-fold to obtain the working bacterial suspension. As a result, the final bacterial concentration in each well was approximately 1.0 × 10^6^ cfu/mL. Bacteria were added to all wells except those in the last row of the plate, which served as the negative control to verify the sterility of the medium and the environmental conditions during the assay. The prepared plates were incubated for 18 h at 36 °C ± 1 °C under monitored conditions. Each MIC assay was performed in triplicate.

### 4.5. MIC Readings

Since many EOs can cause natural turbidity in MHB at high concentrations and may form a dense precipitate at the bottom of the wells after prolonged incubation, MIC readings can be difficult or sometimes impossible, potentially leading to false results. Therefore, after 18 h of incubation, 20 µL of sterile 0.01% resazurin solution (POL-AURA, Olsztyn, Poland) was added to each well of the test plate, followed by an additional 6-h incubation. Resazurin is a dark blue dye that changes to pink only in the presence of live cells, indicating the dilutions of EOs or analytical standards in the wells that are ineffective. MIC was defined as the last dilution of the EOs where the color of the bacterial culture remained unchanged (i.e., blue) after a total of 24 h of incubation. Sample plates are shown in the “Supplementary Material” section (Figure S7).

### 4.6. Statistical Analysis

All experiments were performed in triplicate, and results were statistically analyzed using PQStat software (v. 1.8.4). The chemical composition of EOs is expressed as the mean values of relative content [%] ± standard deviation (SD) of three determinations. MIC values of the three EOs were compared using the Friedman test, followed by Dunn’s post hoc test with Bonferroni correction, and the Page test for trend. A multivariate statistical analysis was performed using PCA. Statistical significance was set at *p* < 0.05.

## 5. Conclusions

The chemical composition of the essential oils (EOs) from the three mint species differed significantly, directly influencing their antibacterial activity. Among them, peppermint oil showed the highest efficacy in inhibiting the growth of all tested *E. coli* strains (MIC 0.5–2.0% *v*/*v*). Menthol, the main component of peppermint oil (approximately 35%), demonstrated even greater activity against both sensitive and resistant strains (MIC 0.125–0.5% *v*/*v*). Therefore, mint oils with a high menthol content appear most promising for controlling pathogenic *E. coli* strains. Based on our results, it can be inferred that the main components of these oils contribute to their overall antibacterial activity. Further studies, including in vivo investigations and research on synergistic interactions with conventional antibiotics and chemotherapeutics, are necessary to fully realize the antibacterial potential of mint EOs against *E. coli* strains.

## Figures and Tables

**Table 1:** Chemical composition of *Mentha* essential oils.

No.	Compound	RT ^1^ (min)	LRI ^2^	LRI_Ref_^3^	Mint Variety		
					*M. piperita* *(Peppermint)*	*M. spicata* *(Spearmint)*	*M. pulegium* *(Pennyroyal)*
					Relative Content ± SD ^4^ (%)		
1	α-Pinene	5.64	932	936	0.89 ± 0.06	0.80 ± 0.06	0.66 ± 0.02
2	Sabinene	6.66	950	969	0.59 ± 0.05	0.60 ± 0.02	0.15 ± 0.01
3	β-Pinene	6.74	975	978	1.17 ± 0.09	1.05 ± 0.02	0.50 ± 0.01
4	β-Myrcene	7.12	990	989	0.22 ± 0.02	1.26 ± 0.04	0.10 ± 0.00
5	3-Octanole	7.28	996	993	0.19 ± 0.01	0.65 ± 0.01	1.02 ± 0.03
6	Limonene	8.23	1027	1030	7.95 ± 0.30	1.93 ± 0.06	1.65 ± 0.02
7	1,8-Cineole	8.36	1031	1032	0.36 ± 0.04	18.73 ± 0.73	0.19 ± 0.01
8	*trans*-β-Ocimene	8.53	1037	1048	0.18 ± 0.02	nd	nd
9	γ-Terpinene	9.16	1057	1060	0.39 ± 0.02	0.55 ± 0.01	nd
10	p-Mentha-3,8-diene	9.51	1068	1068	nd	nd	0.71 ± 0.01
11	*trans*-Menthone	12.33	1155	1150	23.11 ± 0.28	0.40 ± 0.01	6.60 ± 0.10
12	Menthofuran	12.72	1167	1159	4.87 ± 0.20	nd	nd
13	*cis*-Menthone	12.73	1167	1159	nd	nd	1.64 ± 0.05
14	neo-Menthol	12.80	1169	1167	6.80 ± 0.23	nd	nd
15	Menthol	13.02	1176	1177	35.14 ± 0.06	1.83 ± 0.07	nd
16	*trans*-Isopulegone	13.05	1177	1177	nd	nd	2.98 ± 0.11
17	Terpinen-4-ol	13.08	1178	1177	nd	1.27 ± 0.05	nd
18	Isomenthol	13.47	1189	1179	0.60 ± 0.10	nd	nd
19	α-Terpineol	13.68	1196	1190	0.57 ± 0.04	nd	nd
20	*cis*-Dihydrocarvone	13.75	1198	1191	nd	3.09 ± 0.02	nd
21	*trans*-Dihydrocarvone	13.96	1204	1201	nd	1.03 ± 0.09	nd
22	Pulegone	15.42	1248	1234	1.58 ± 0.01	nd	76.54 ± 0.65
23	Carvone	15.57	1253	1242	nd	58.61 ± 0.51	nd
24	Piperitone	15.95	1264	1254	0.57 ± 0.01	0.70 ± 0.20	nd
25	Neomenthyl acetate	16.33	1276	1271	0.45 ± 0.01	nd	nd
25	Menthyl acetate	17.05	1298	1296	6.96 ± 0.12	0.48 ± 0.02	nd
27	*cis*-Dihydrocarvyl acetate	18.10	1330	1326	nd	0.50 ± 0.01	nd
28	Piperitenone	18.50	1343	1341	nd	nd	1.98 ± 0.16
29	Carvyl acetate	19.18	1364	1362	nd	0.34 ± 0.01	nd
30	β-Bourbonene	19.85	1385	1384	0.23 ± 0.00	2.18 ± 0.06	nd
31	β-Elemene	20.09	1393	1390	nd	0.26 ± 0.02	nd
32	β-Caryophyllene	20.95	1420	1420	3.55 ± 0.23	1.74 ± 0.05	1.44 ± 0.05
33	α-Humulene	21.95	1453	1453	0.17 ± 0.01	0.35 ± 0.02	1.91 ± 0.09
34	β-Farnesene	22.09	1457	1457	0.27 ± 0.01	nd	nd
35	Germacrene D	22.81	1481	1481	1.25 ± 0.02	nd	0.15 ± 0.01
36	Bicyclogermacrene	23.27	1496	1494	0.27 ± 0.01	nd	nd
Total					98.43	98.38	98.24

^1^ RT—retention time; ^2^ LRI—linear retention index determined experimentally in relation to n-alkanes (C_7_–C_30_) on HP5-MSI column; ^3^ LRI_Ref_—reference linear retention index from the literature, ^4^ SD—standard deviation (*n* = 3); nd—not detected.

**Table 2:** Susceptibility of *Escherichia coli* strains isolated from poultry to essential oils (EOs) from various *Mentha* species and their main constituents, as determined by the minimum inhibitory concentration (MIC).

*Escherichia coli* Strain		MIC ^1^ of Main Constituents or Essential Oil (% *v*/*v*)						
		Menthol	Menthone	PM EO ^2^	Carvone	SM EO ^3^	Pulegone	PR EO ^4^
Multidrug-resistant strains								
1		0.125	2	1	2	2	2	4
2		0.25	8	1	4	4	4	4
3		0.25	8	2	4	4	4	4
4		0.25	8	1	4	4	4	4
5		0.125	4	0.5	2	2	1	4
6		0.25	4	1	2	4	2	4
7		0.25	8	1	2	2	2	4
8		0.5	8	2	4	4	4	4
9		0.5	8	2	4	4	2	4
10		0.125	8	1	4	4	2	4
Susceptible strains								
11		0.125	4	0.5	2	4	2	4
12		0.125	2	0.5	2	2	2	4
13		0.125	2	0.5	2	2	2	4
14		0.125	2	0.5	2	2	2	4
15		0.25	8	1	4	4	4	4
16		0.25	2	0.5	2	2	2	4
17		0.125	2	1	2	4	4	4
18		0.125	1	0.5	2	2	2	4
19		0.125	4	2	4	4	4	4
ATCC 25922		0.125	2	0.5	2	2	2	4
Summary								
Mean MIC		0.18	3.86	0.87	2.64	2.93	2.46	4
SD ^5^		0.12	2.85	0.56	1	1	1	0
CV ^6^ %		66.7	73.8	64.4	37.9	34.1	40.7	0
most frequent MICs	% *v*/*v*	0.125	8	0.5/1	2	4	2	4
	% of cases	55	40	40/40	60	55	60	100

^1^ MIC—minimal inhibitory concentration ^2^ MP EO—peppermint essential oil; ^3^ SM EO—spearmint essential oil; ^4^ PR EO—pennyroyal essential oil; ^5^ SD—standard deviation; ^6^ CV—coefficient of variation.

**Table 3:** Statistical analysis for results of minimum inhibitory concentration (MIC).

Group		Total			Multidrug-Resistant Strains			Susceptible Strains		
		PM EO ^2^	SM EO ^3^	PM EO ^4^	PM EO ^2^	SM EO ^3^	PR EO ^4^	PM EO ^2^	SM EO ^3^	PR EO ^4^
		(1)	(2)	(3)	(1)	(2)	(3)	(1)	(2)	(3)
Number of strains		20	20	20	10	10	10	10	10	10
Arithmetic mean		1	3.1	4	1.25	3.4	4	0.75	2.8	4
Standard deviation		0.56	1.02	0	0.5401	0.9661	0	0.4859	1.0328	0
Friedman test	T1	37.1304			18.7273			18.6667		
	*p*	<0.0001			0.0001			0.0001		
Post hoc ^1^	(1)		<0.0001	0.0002		0.0007	0.0076		0.0002	0.0219
	(2)	0.0002	0.4642		0.0076	1		0.0219	0.5391	
	(3)	<0.0001		0.4642	0.0007		1	0.0002		0.5391
Homogeneous groups		a	b	b	a	b	b	a	b	b
Test page for trend	Z	4.0319			3.0187			2.6833		
	*p*	0.0001			0.0025			0.0073		

^1^ Dunn Bonferroni; ^2^ MP EO—peppermint essential oil; ^3^ SM EO—spearmint essential oil; ^4^ PR EO—pennyroyal essential oil.

## Data Availability

The original contributions presented in this study are included in the article/Supplementary Material. Further inquiries can be directed to the corresponding author.

## Supplementary Materials

The following supporting information can be downloaded at: https://www.mdpi.com/article/10.3390/ijms262311263/s1.

## Abbreviations

The following abbreviations are used in this manuscript:

APEC	Avian Pathogenic *Escherichia coli*
EO	Essential oil
EOs	Essential oils
MIC	Minimum inhibitory concentration
