# Supplementary material for: Potential of Essential Oils from Different Mint Species Against Multidrug-Resistant Escherichia coli Strains Isolated from Clinical Cases in Poultry
Source: Int J Mol Sci. 2025 Nov 21;26(23):11263. doi: 10.3390/ijms262311263 (PMC12691715; doi:10.3390/ijms262311263)
Supplement: Supplementary file 1 [file ijms-26-11263-s001.zip › ijms-3942222-supplementary.pdf]

Supplementary Material

Supplementary Figure S1. Total Ion Chromatogram of *Mentha piperita* (peppermint) essential oil obtained by GC-MS method.

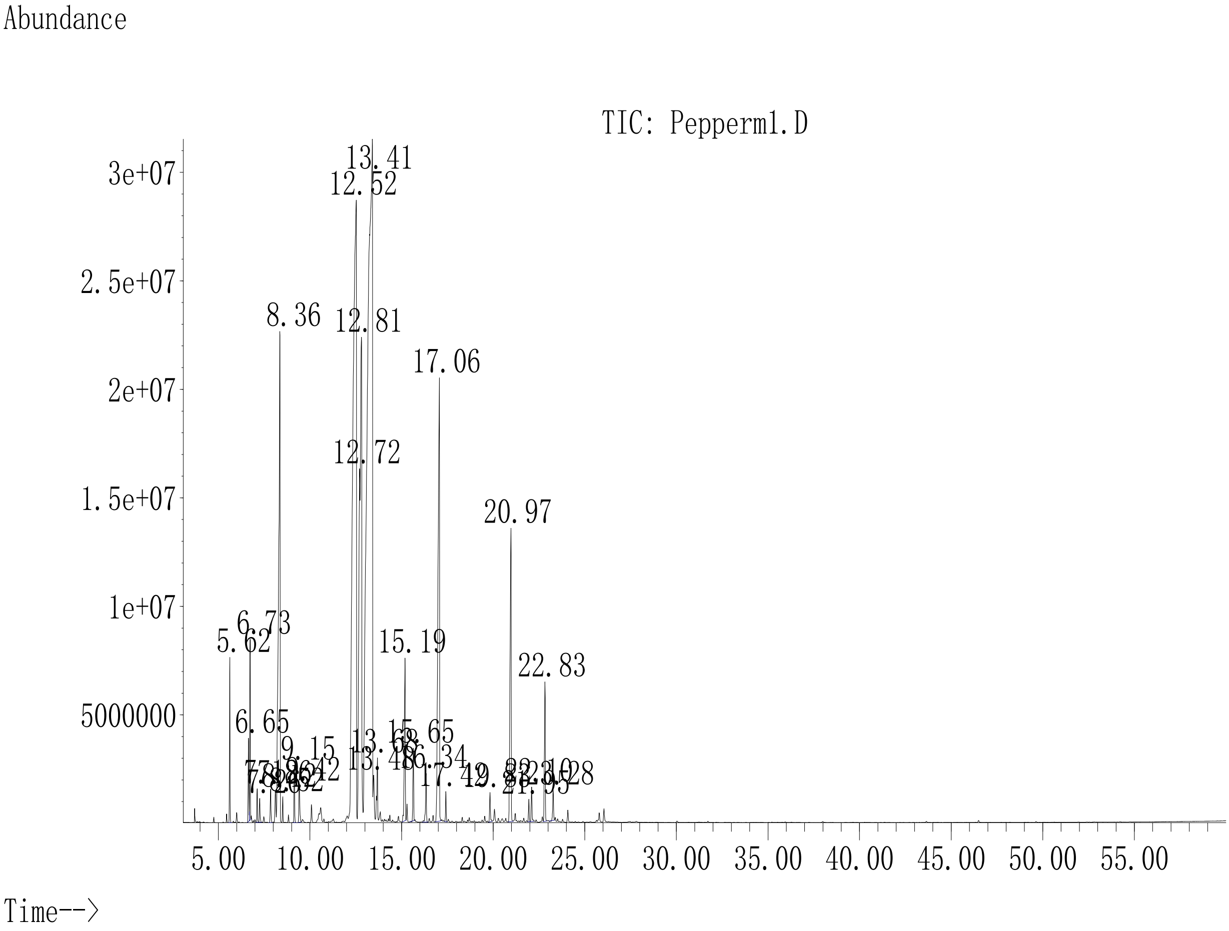

Supplementary Figure S2. Mass spectrum of menthol present in *Mentha piperita* (peppermint) essential oil, compared with menthol standard mass spectrum from NIST 04 library.

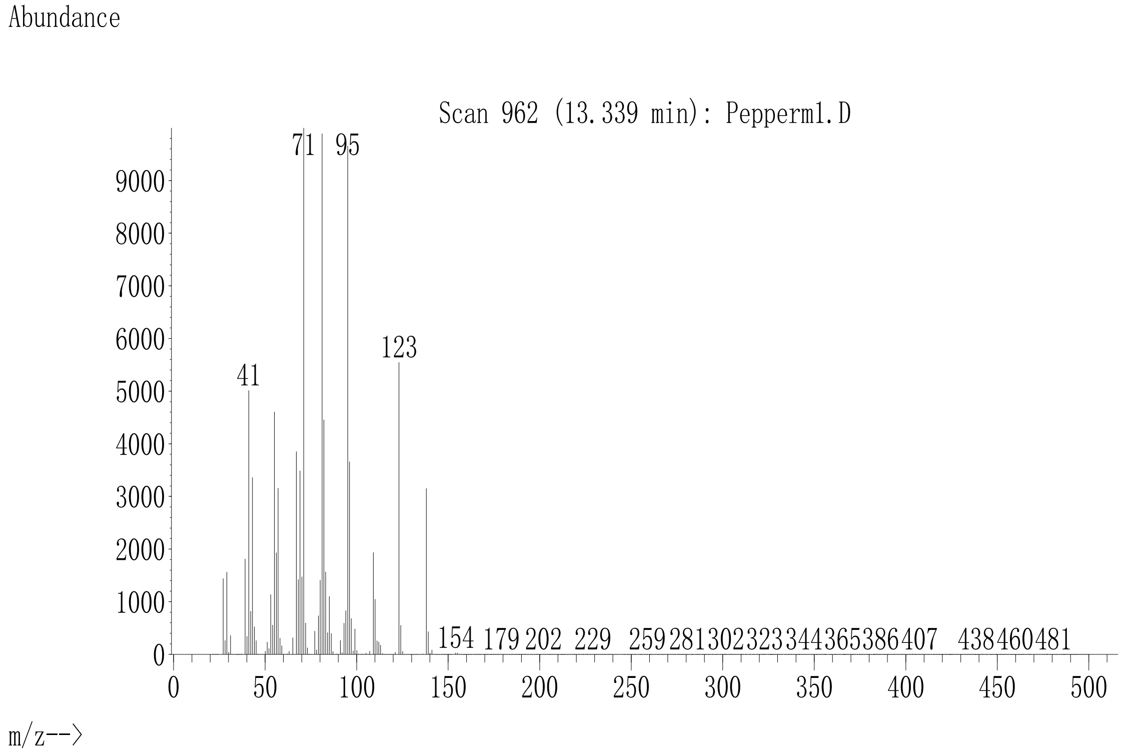

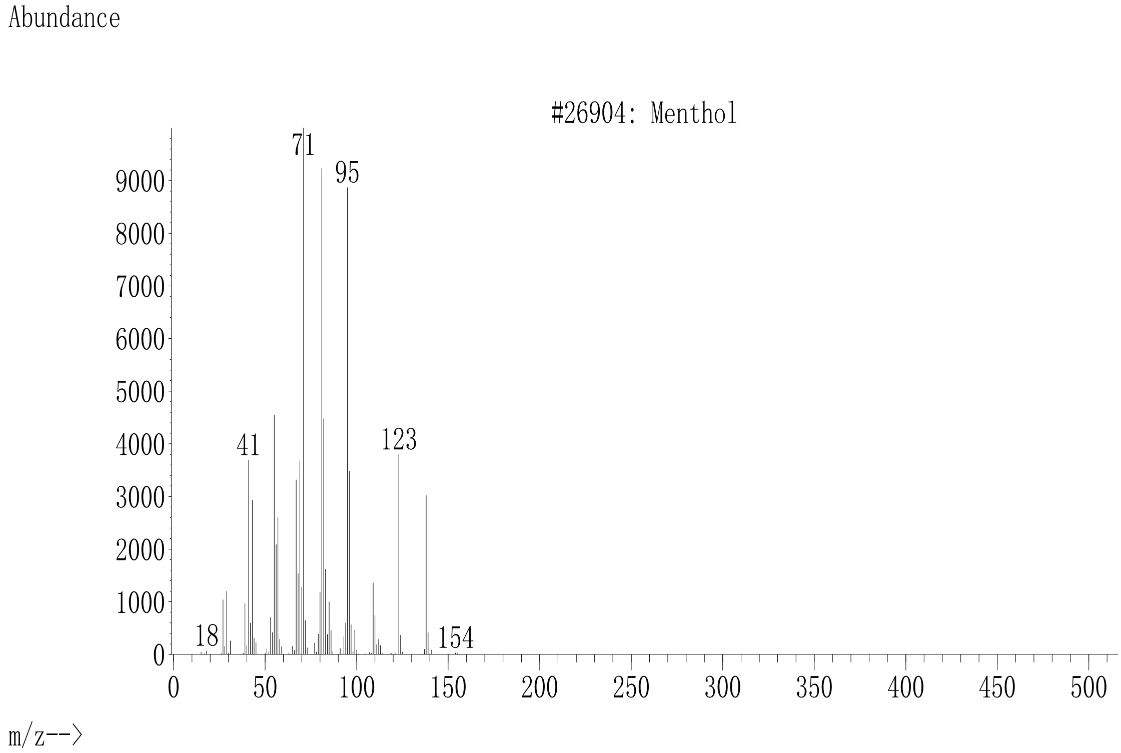

Supplementary Figure S3. Total Ion Chromatogram of *Mentha spicata* (spearmint) essential oil obtained by GC-MS method.

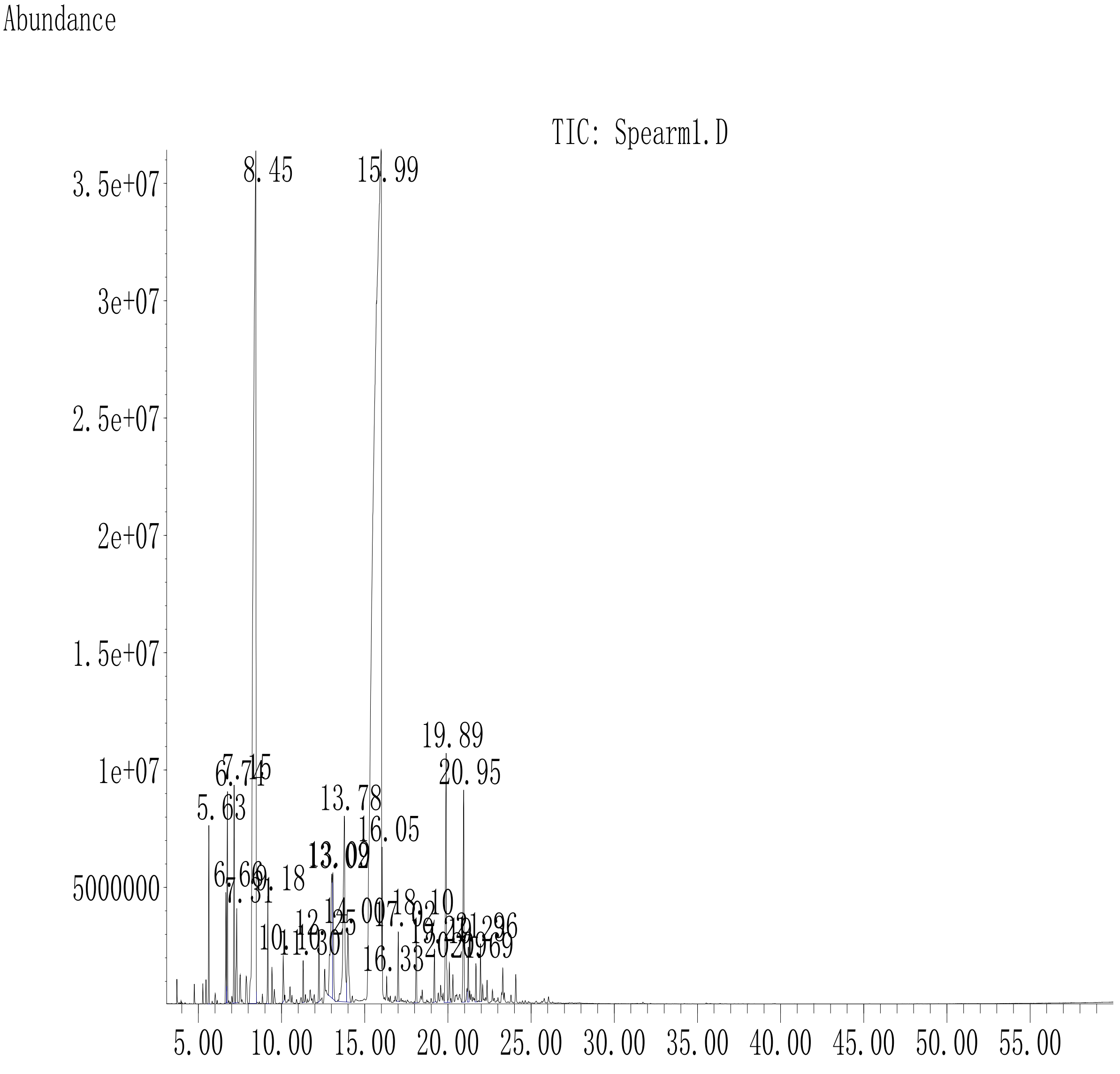

Supplementary Figure S4. Mass spectrum of carvone present in *Mentha spicata* (spearmint) essential oil, compared with carvone standard mass spectrum from NIST 04 library.

Abundance

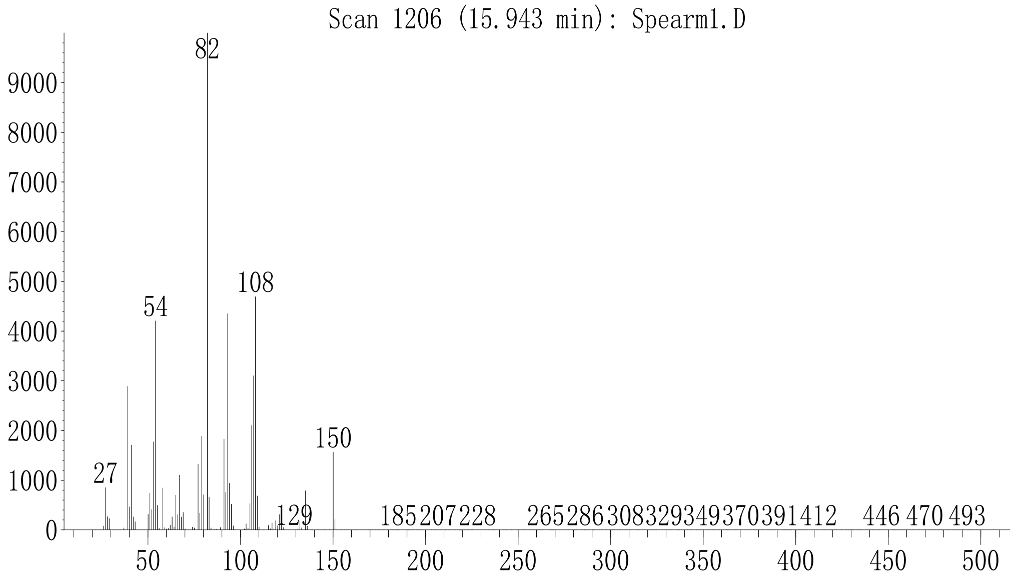

m/z-->
Abundance

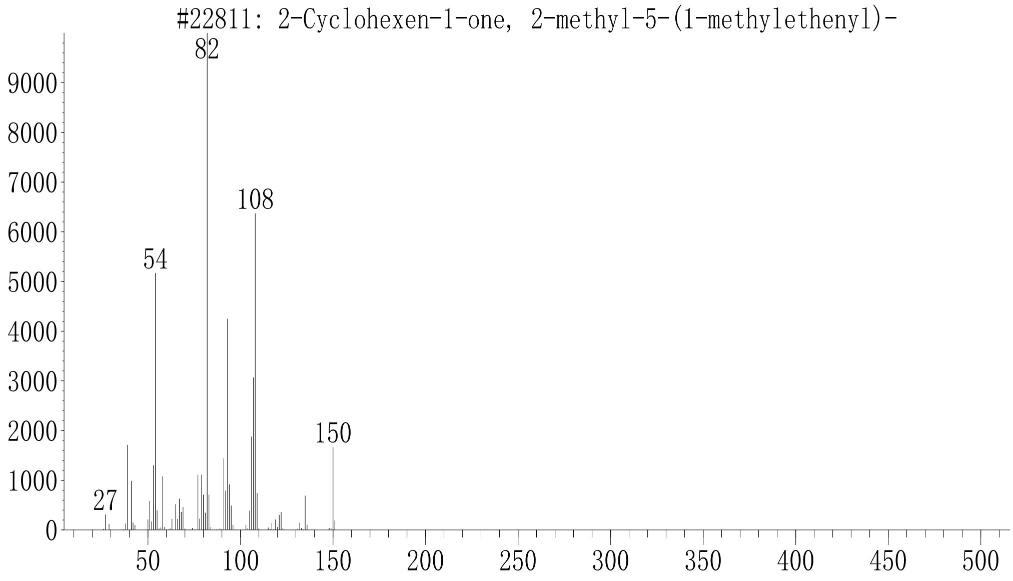

m/z-->

Supplementary Figure S5. Total Ion Chromatogram of *Mentha pulegium* (pennyroyal) essential oil obtained by GC-MS method.

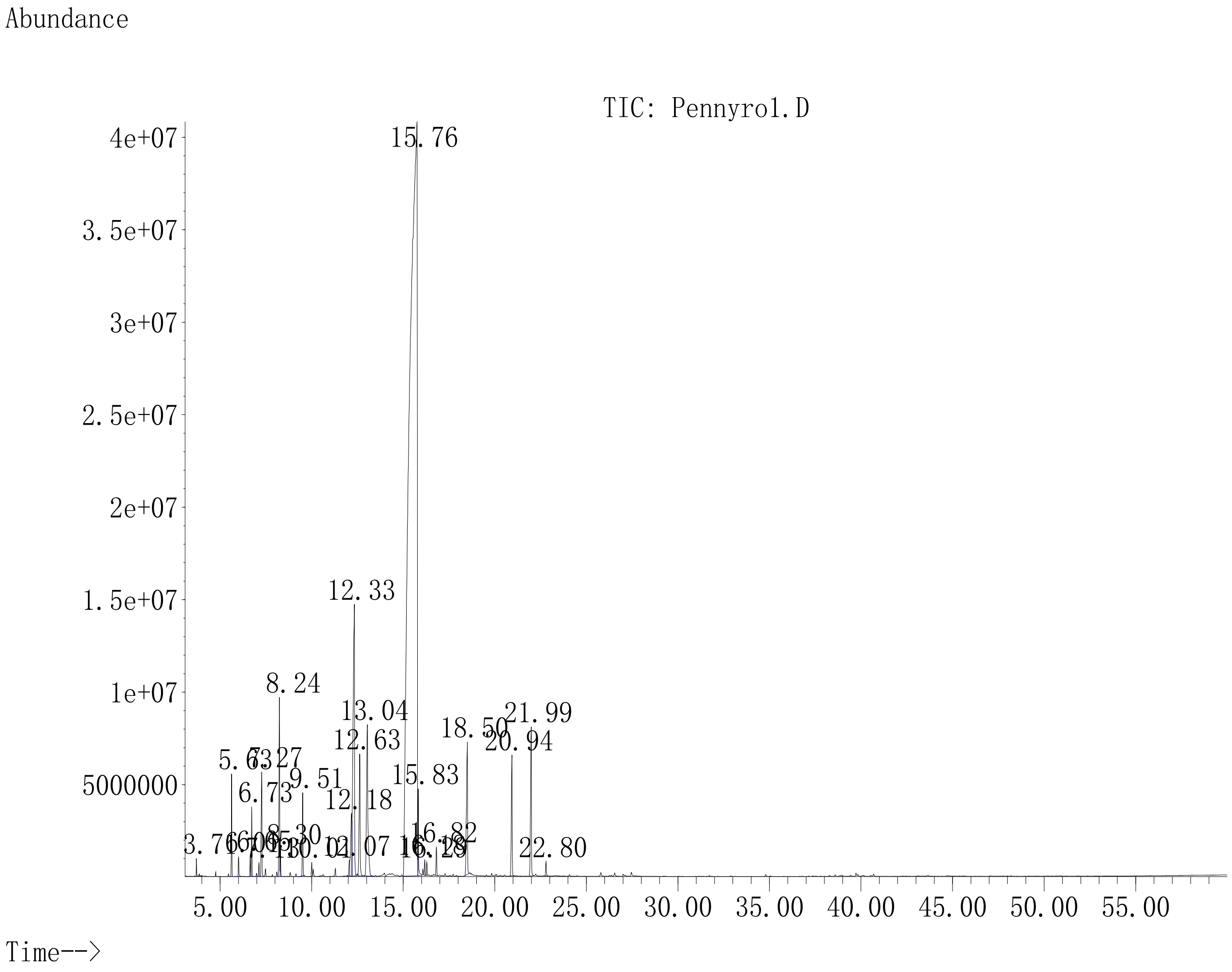

Supplementary Figure S6. Mass spectrum of pulegone present in *Mentha pulegium* (pennyroyal) essential oil, compared with pulegone standard mass spectrum from NIST 04 library

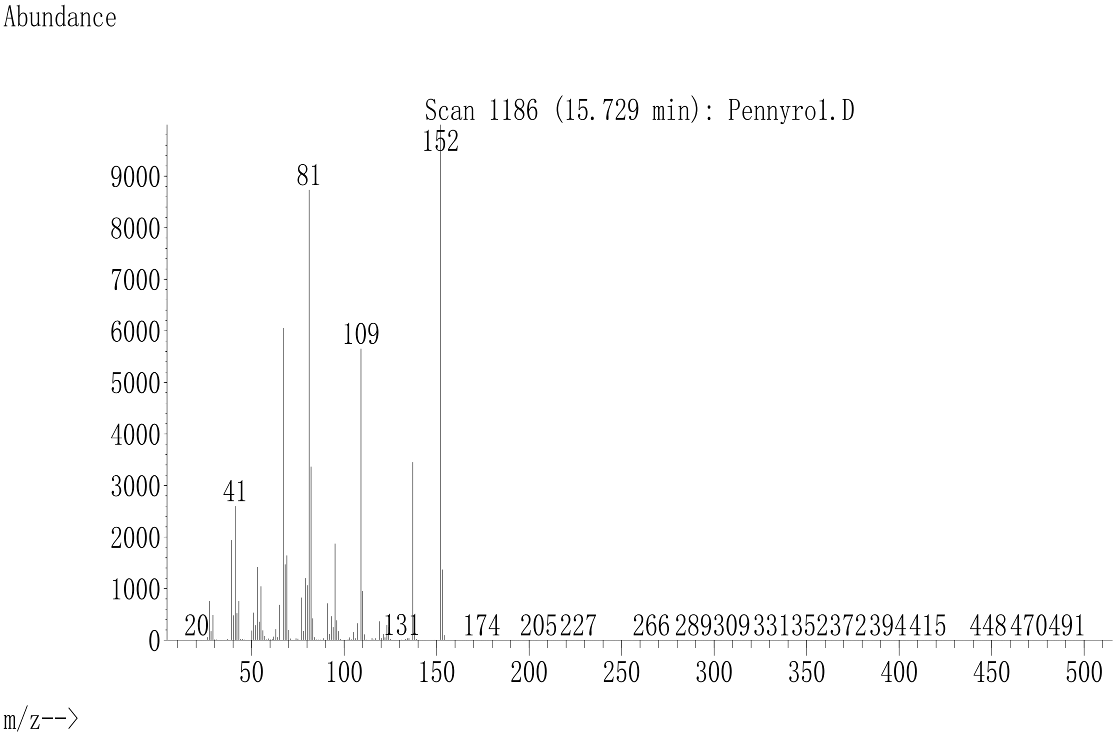

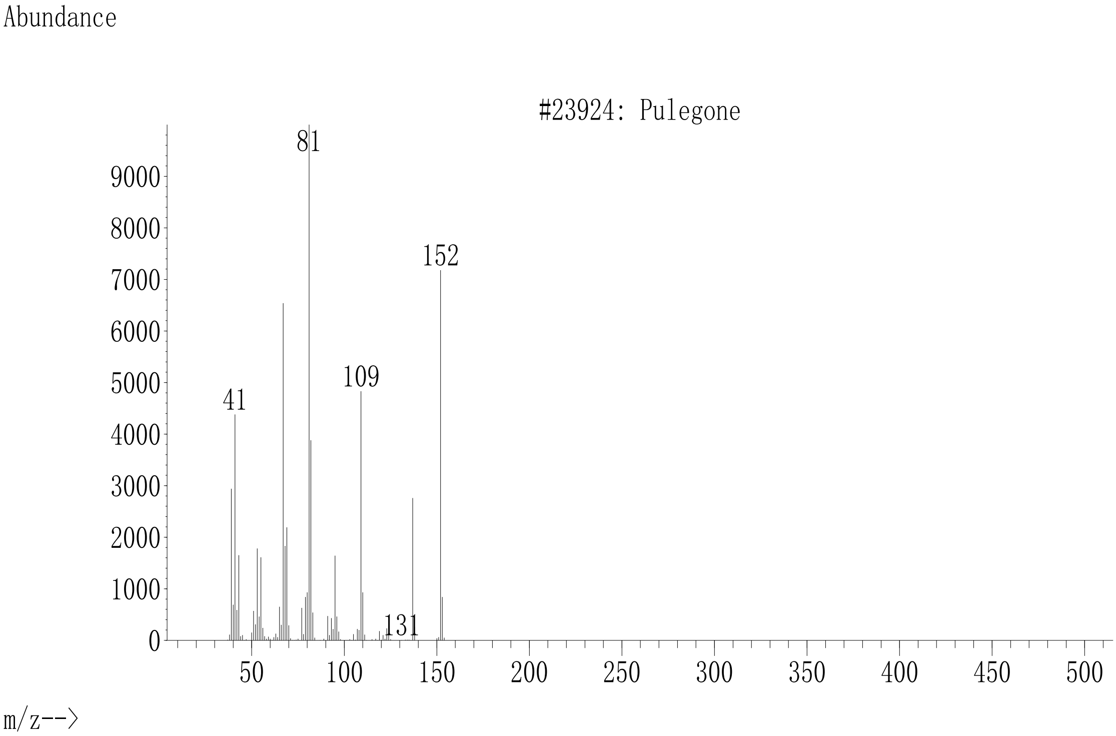

Supplementary Figure S7. Example of plates after a total of 24 hours of incubation and addition of resazurin for the drug-resistant (No. 6) and the antibiotic-sensitive (ATCC 25922) strains.

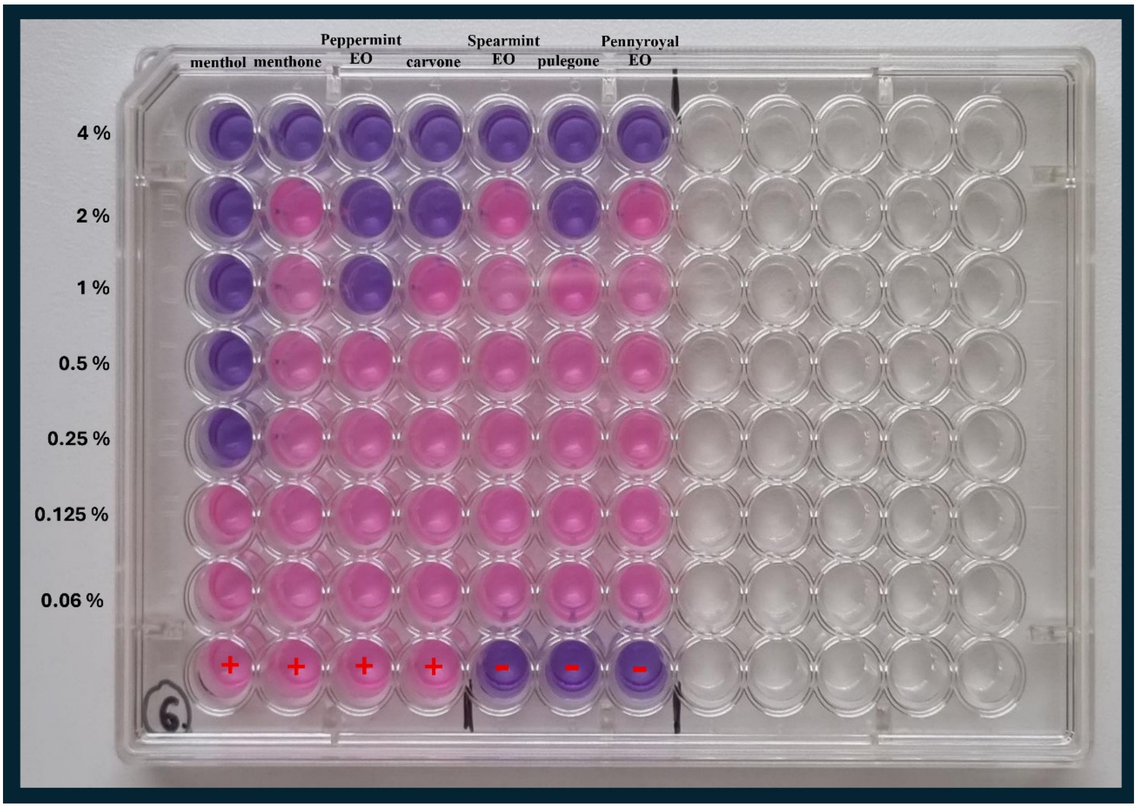

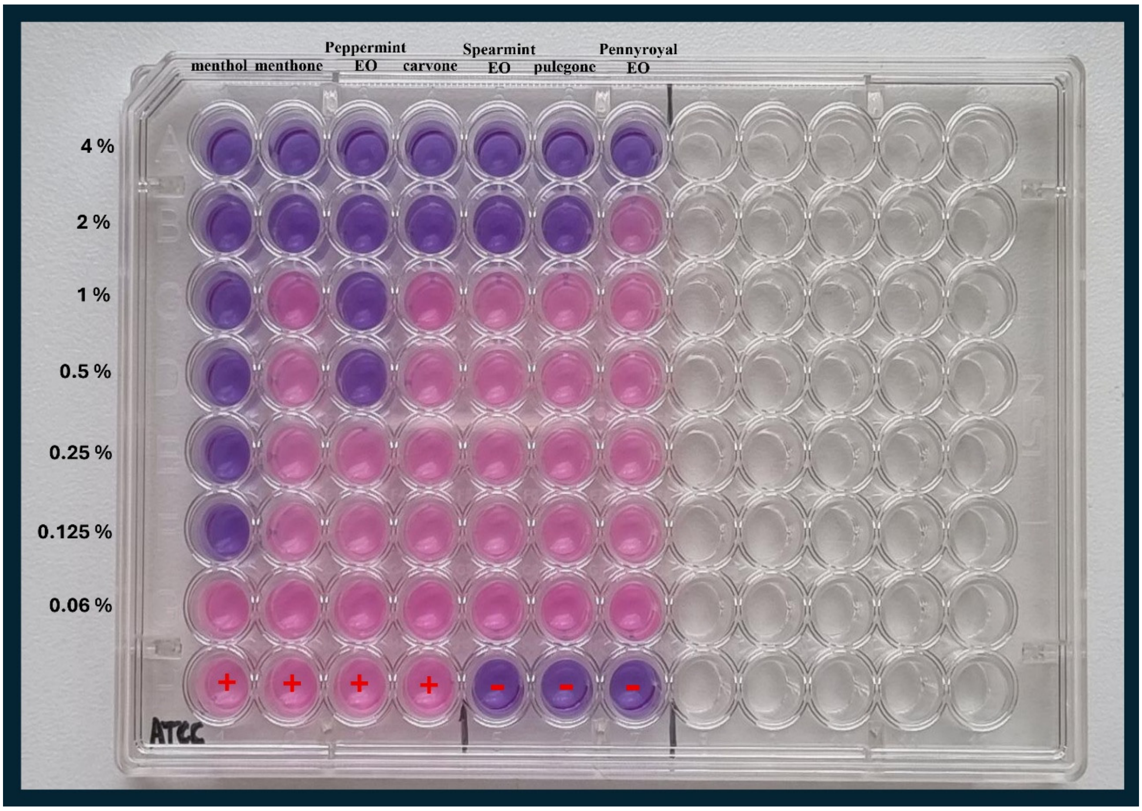

Supplementary Table S1. Drug susceptibility of selected isolates of *Escherichia coli* (n=19) and the ATCC 25922 reference strain (source: official test reports; disk-diffusion method)

No.	*E. coli* strain No.	antimicrobial agent								
		AMX	AMC	ENR	SXT	DO	OT	FFC	CT	LS
1.	1	R	R	R	R	R	R	R	S	R
2.	2	R	R	R	R	R	R	R	S	R
3.	3	R	S	R	R	R	R	R	S	R
4.	4	R	R	R	R	R	R	S	S	R
5.	5	R	R	R	R	R	R	S	S	R
6.	6	R	S	R	R	R	R	R	S	S
7.	7	R	S	R	R	R	R	S	S	R
8.	8	R	S	R	R	R	R	S	S	S
9.	9	R	S	R	R	R	R	S	S	S
10.	10	R	S	R	R	R	R	S	S	S
11.	11	R	S	I	S	I	R	S	S	S
12.	12	R	S	I	R	S	S	S	S	S
13.	13	R	S	S	S	S	S	S	S	S
14.	14	S	S	S	S	S	S	S	S	S
15.	15	S	S	S	S	S	S	S	S	S
16.	16	S	S	S	S	S	S	S	S	S
17.	17	S	S	S	S	S	S	S	S	S
18.	18	S	S	S	S	S	S	S	S	S
19.	19	S	S	S	S	S	S	S	S	S
20.	ATCC 25922	S	S	S	S	S	S	S	S	S

R resistant   I intermediate   S susceptible

AMX – amoxicillin
AMC – amoxicillin/clavulanic acid
ENR – enrofloxacin
SXT – sulfamethoxazole/trimethoprim
DO – doxycycline
OT – oxytetracycline
FFC – florfenicol
CT – colistin
LS – lincomycin/spectinomycin

Supplementary Table S2. Susceptibility of *E. coli* strains isolated from poultry to essential oils (EOs) from various *Mentha* species and their main constituents, as determined by the minimum inhibitory concentration (MIC).

***Escherichia coli* strain**	**MIC of main constituents or essential oil (mg/mL)**						
	**menthol**	**menthone**	**Peppermint**	**carvone**	**Spearmint**	**pulegone**	**Pennyroyal**
**Multidrug-resistant strains**							
1	1.1	17.9	9	19.2	18.5	18.7	37.1
2	2.2	71.6	9	38.4	37	37.4	37.1
3	2.2	71.6	18	38.4	37	37.4	37.1
4	2.2	71.6	9	38.4	37	37.4	37.1
5	1.1	35.8	4.5	19.2	18.5	9.3	37.1
6	2.2	35.8	9	19.2	37	18.7	37.1
7	2.2	71.6	9	19.2	18.5	18.7	37.1
8	4.4	71.6	18	38.4	37	37.4	37.1
9	4.4	71.6	18	38.4	37	18.7	37.1
10	1.1	71.6	9	38.4	37	18.7	37.1
**Susceptible strains**							
11	1.1	35.8	4.5	19.2	37	18.7	37.1
12	1.1	17.9	4.5	19.2	18.5	18.7	37.1
13	1.1	17.9	4.5	19.2	18.5	18.7	37.1
14	1.1	17.9	4.5	19.2	18.5	18.7	37.1
15	2.2	71.6	9	38.4	37	37.4	37.1
16	2.2	17.9	4.5	19.2	18.5	18.7	37.1
17	1.1	17.9	9	19.2	37	37.4	37.1
18	1.1	8.9	4.5	19.2	18.5	18.7	37.1
19	1.1	35.8	18	38.4	37	37.4	37.1
ATCC 25922	1.1	17.9	4.5	19.2	18.5	18.7	37.1
Density (g/mL)	0.890	0.896	0.901	0.960	0.924	0.935	0.927
